# Isolation and characterization of *Saprolegnia parasitica* from cage-reared *Pangasianodon hypophthalmus* and its sensitivity to different antifungal compounds

**DOI:** 10.1038/s41598-024-80075-0

**Published:** 2024-12-28

**Authors:** Sanjaykumar Karsanbhai Rathod, Basanta Kumar Das, Ritesh Shantilal Tandel, Sohini Chatterjee, Nilemesh Das, Gayatri Tripathi, Saurav Kumar, Satyen Kumar Panda, Prasanna Kumar Patil, Sanjib Kumar Manna

**Affiliations:** 1 ICAR-Central Institute of Fisheries Education, Mumbai, 400061 India; 2 ICAR-Central Inland Fisheries Research Institute, Kolkata, 700120 India; 3 ICAR-Central Institute of Brackishwater Aquaculture, Navsari, 396450 India; 4 Food Safety and Standard Authority of India, New Delhi, 110002 India; 5 ICAR-Central Institute of Brackishwater Aquaculture, Chennai, 600028 India

**Keywords:** *Pangasianodon hypophthlamus*, *Saprolegnia parasitica*, Cage culture, Antifungal sensitivity, Temperature, Drug discovery, Microbiology, Zoology

## Abstract

Saprolegniasis is one of the most dangerous fungal diseases of fish, causing significant mortality in fish hatcheries and young ones. The present study aimed to isolate and characterize the causative fungus from fingerlings of *Pangasianodon hypophthalmus* cultured intensively in freshwater cages in Indian reservoirs and to determine minimum inhibitory concentrations of different antifungal compounds against the fungal hyphae and zoospores. The fungal isolates grown on potato dextrose agar showed an abundance of gemmae, elongated mycelia, non-septate hyphae, primary zoospores, mature zoosporangia with numerous zoospores, cysts with bundles of long hairs and were further identified as *Saprolegnia parasitica* following PCR amplification and sequencing of internal transcribed spacer region. *S. parasitica* showed temperature-sensitive optimum growth in a narrow window of 12–24 ℃, which might drive its experimental pathogenesis as well as natural infections in the winter months. In vitro sensitivity testing established negligible inhibitory activity of fluconazole, boric acid, sodium thiosulfate, and potassium permanganate while clotrimazole arrested the spore and hyphal growths at 2 mgL^-1^ concentration suggesting potential of the imidazole antifungal in treating *S. parasitica* infection in fish. The present study will serve as the baseline information for developing therapeutic and management strategies for controlling saprolegniasis in the economically significant iridescent catfish.

## Introduction

In the last two decades, the culture of iridescent catfish *Pangasianodon hypophthalmus*, commonly known as Pangasius, has increased by more than 22 folds, making it one of the most sought-after aquaculture products worldwide. Pangasius contributed 5.1% to the global inland finfish aquaculture production (FAO, 2022), 40% of the global catfish production, and has a projected growth rate of 4.3% in the ASEAN region during 2015–30^1^. Vietnam is the major catfish producer, generating about 1.7 million tonnes by 2021, whereas India produced 0.55 million tonnes during 2021–22 (Department of Fisheries, 2022). It is India’s third most cultivable fish species, after *Labeo rohita* and *L. catla*. Besides high productivity in tanks, Pangasius is cultured in very high densities in freshwater cages in reservoirs and floodplain wetlands of India with an average yield of 30.4 kg m^−3^ cage area (pers. comm.).

Despite consistently high growth rate of the aquaculture sector in last few decades, disease outbreaks have remained a significant challenge, especially in intensive farming systems. Superficial mycosis, caused by *Saprolegnia parasitica* and *S. declina* complex, is one of the major fungal diseases of fish worldwide. Various fish species including *Oreochromis niloticus*^2^, *P. hypophthalmus*^3^, *Schizothorax richardsonii* (Tandel et al.^4^), *Clarias batrachus*^5^, Indian Major Carps^6^, *Channa* spp.^7^, Mullet, Tench, Barramundi and Sturgeon^8^ are significantly affected by *Saprolegnia*. Low water temperature and survival of the secondary cyst in the culture system favour the development of saprolegniasis in fish (Tandel et al.^4^). Viable zoospores of *S. parasitica* colonize the body surface of fish, impair osmoregulation, and cause tissue damage through secreted enzymes^3,9,10^. Several putative proteins, including CBD proteins, CBEL-like proteins, glycosyl hydrolases, proteases, and protease inhibitors contribute to virulence of the fungus^11,12^. Salmon farms and hatcheries in Scotland, Norway, and Chile incur more than 10% economic loss due to *Saprolegnia* infections^13^. In Japan, *S. parasitica* can cause up to 50% mortality in Coho salmon and eel^14^. The catfish industry in the United States loses about $50 million annually from *Saprolegnia* infection^15^.

Occurrence of superficial mycosis in freshwater cage culture was reported by Bera et al.^9^, but no systematic study has been conducted on identification of the causative agent. Despite significant loss from the disease, there are meagre works on developing preventive and therapeutic measures against the water mould. Antimicrobial sanitizers such as iodine, formalin, hydrogen peroxide, boric acid, sodium thiosulfate, immunostimulants, etc. are used to contain fungal outbreaks^5,14,16–18^, however, there is not enough convincing information about effectiveness of these chemicals against various life stages of *Saprolegnia*. The present study has identified the aetiological agent of superficial mycosis in Pangasius cultured in cages. Sensitivity of the fungus to some common chemicals and antifungal drugs have been investigated to identify a potential therapeutic agent.

## Materials and methods

### Disease survey in freshwater cage culture and oomycete isolation

In the present study, fish cage enclosures were surveyed in different reservoirs of Jharkhand and Chhattisgarh, two major freshwater cage culture states of India, for the occurrence of fungal diseases. Fingerlings and growing stages of Pangasius with surface lesions such as ulcers and cotton wool-like growths were segregated and humanely euthanized by overdoses of MS222 (HiMedia, India), collected in sterile plastic bags, and transported to the laboratory under ice cover. Fish tails, fins or body surfaces with suspected fungal growths and ulcerated skin lesions were disinfected with 70% ethanol for a few seconds and then washed with sterile distilled water three to four times. Surface lesions with body parts were taken out aseptically using a tissue scrapper and placed onto Sabourad dextrose agar (SDA, HiMedia, India), inhibitory mould agar (IMA, HiMedia, India), peptone yeast extract agar (PYEA, HiMedia, India), and potato dextrose agar (PDA, HiMedia, India) containing chloramphenicol (50–125 mgL^−1^) following Tandel et al.^19^. The plates were incubated at 18 ℃ for a week and examined for fungal growth, which was sub-cultured a few times for isolation in pure culture.

### Morphological identification

A tentative identification of the fungus isolates as *S. parasitica* was made based on their growth and morphological characteristics following Tandel et al.^19^ with minor modifications. Freshly grown hyphae edges, about 5 mm in diameter, were excised out from a culture plate and placed in middle of new PDA plates and autoclave-sterilized sesame seeds were placed at the periphery of the plates as baits for zoospore germination. Following incubation at 18 ℃ for 48 h, sesame seeds with attached hyphae were harvested, washed twice in sterile water and transferred to autoclaved pond water (APW) in 90 mm sterile petri plates. Water from a nearby aquaculture pond was collected and autoclave sterilized to make APW. The fungal growths were then examined under an inverted microscope (Zeiss, Axioskop2, Germany) for hypha and zoospore formations and their structures. The study was repeated for 3 weeks for re-emergence of zoospores.

### Scanning electron microscopy of fungal morphology

Scanning electron microscope was applied for a magnified view of the fungal isolates following Rezinciuc et al.^20^. A fresh hyphal growth of about 5 mm diameter was placed on PDA, and sesame seeds were inoculated around the inoculum. Following incubation for 72 h at 18 ℃, the sesame seeds (with attached hyphae) were washed twice with phosphate-buffered saline (PBS, pH 7.2), kept in APW and incubated at 18 ℃ for 96 h when the freshly grown hyphae on sesame seeds were taken out, washed with distilled water and PBS and fixed with 2.5% glutaraldehyde at 4 ℃ for 30 min. These were then dehydrated in 30%, 50%, 70%, 90% and 100% alcohol for 3 min each, followed by final air drying at room temperature. The samples were then coated with metallic gold particles of approximately 80 nm diameter in a vacuum coater and observed under a scanning electron microscope (Hitachi S-530) at accelerating voltages of 15 and 20 kV for detailed morphology.

### Molecular identification and phylogenetic analysis

Pure oomycete cultures were inoculated in potato dextrose broth and grown at 20 ℃ for 72 h. After incubation, fungal hyphae with clumps were taken out and washed twice with PBS in a 2 mL centrifuge tube. Fungal DNA was extracted using a commercial kit (Genetix, Nucleopore, India) following manufacturer’s instructions. The extracted DNA preparations were quantified in a fluorometer (Qubit 3.0; Life Technologies). For molecular identification, the internal transcribed spacer (ITS) region of the fungus was amplified using universal primers ITS 1: TCCGTAGGTGAACCTGCGG and ITS 4: TCCTCCGCTTATTGATATGC^21^. The PCR reaction was conducted in 25 μL volume containing 1 μL of each primer (10 pmol μL^−1^), 2 μL genomic DNA, 12 μL master mixture (IDT, USA) and 9 μL nuclease-free water (Sigma, USA) in a thermal cycler (Biorad, T100). The reaction profile included initial denaturation at 94 °C for 2 min, 35 cycles of amplification (95 °C for 30 s, 58 °C for 30 s, 72 °C for 1 min) and final elongation at 72 °C for 5 min^4^. The PCR amplicons were separated in 1.5% agarose by gel electrophoresis, purified using a QIAquick® gel extraction kit (Qiagen, Germany) and sequenced by the Sanger method^21^. Qualities of the nucleotide sequence chromatograms were examined using Sequence Scanner v1.0 software (Applied Biosystems, Inc.) and the forward and reverse sequences were aligned using CodonCode Aligner software (CodonCode Corporation) to make contigs, which were BLASTed in NCBI GenBank to find closet phylogenetic affiliations. A phylogenetic tree of the isolate, based on the ITS region sequences, with type strains and closest matches, was prepared by applying MEGA 11 software^3^.

### Study of fungal growth at different temperatures

The optimum temperature requirements of the fungal isolates were examined by culturing on PDA at different temperatures. About 5 mm diameter of hyphal edges of pure cultures were exercised out and placed at the centre of PDA plates in triplicate and incubated at 4, 8, 12, 16, 20, 24, and 28 °C for 72 h. The growth of hypha was measured every 24 h.

### Determination of minimum inhibitory concentration (MIC) of different compounds against zoospores

Antifungal activity of fluconazole (Sigma, USA), clotrimazole (Sigma, USA), potassium permanganate (Merck, Germany), copper sulfate (Merck, Germany), sodium thiosulfate (Merck, Germany), boric acid (HiMedia, India), and malachite green (Merck, Germany) were examined against the fungal isolates. To assess activity against zoospores, zoospores were germinated following the method of Tandel et al.^4^. Briefly, freshly grown hyphae of about 5 mm diameters were placed on PDA plates and surrounded by sesame seeds as bait. Following 48 h incubation, the seed baits with attached hyphae were taken out and washed twice with PBS on sterile glass slides, and then put in 6-well plates containing APW. Following incubation at 20 °C for 18 h, the concentration of zoospores in each well was determined by mixing 100 µL of the APW culture broth with 100 µL of resazurin (HiMedia, India) solution; 10 µL of it was put in a Neubauer haemocytometer and unstained zoospores were counted under a microscope. Then, viable zoospores (5.0–5.2 × 103 nos.mL^−1^) were harvested from the APW.

To determine MICs, 1000 mgL^-1^ concentration of each test chemical was prepared by dissolving in distilled water and sterilization by membrane filtration (0.22 µm filter); fluconazole and clotrimazole were dissolved and diluted in dimethylsulfoxide (DMSO) (Merck, Germany). The highest concentration (1000 mgL^−1^) of each compound was added to 1st well, in triplicates, in a 96-well plate containing an equal volume of glucose yeast broth (HiMedia, India) and then serially diluted up to the 10th well. The 11th and 12th wells served as negative control (only the medium); malachite green was similarly tested and served as the positive control. Then, viable zoospores from the APW plate were added to each of the 96 wells @ 5–5.1 × 10^3^ nos. per well and incubated at 20 ℃ for 18 h, followed by addition of 5 µL of resazurin dye in each well and observation by the naked eye for colour change for up to 2 h. The lowest concentration of a test compound, giving no change of color of resazurin, was taken as its MIC.

### Determination of MIC of different compounds against hyphae

The inhibition of mycelial growth by the chemicals, mentioned above, was tested in PDA supplemented with different concentrations of the test chemicals, following Paria et al.^18^. Stock solutions of the test compounds were prepared at 1000 mgL^-1^, and fungal growth inhibition in PDA was examined at final concentrations of 1–100 mgL^-1^; a narrow range of concentrations between 1 and 10 mgL^-1^ were subsequently used to determine exact MIC of chemicals. Malachite green served as a positive control, and the negative control contained a medium devoid of any antifungal agent. Hyphae from freshly grown colonies were inoculated in the respective plates and incubated at 20 ℃ for 72 h. The hyphal growth was measured at different intervals, and the inhibition rate was calculated using the formula:

$$\% Inhibition Rate=\frac{X-Y}{X-Z} \times 100$$

X is the hyphal growth in control, Y is the hyphal growth in the test chemical, and Z is the average diameter of hyphal growth.

### Experimental pathogenicity study

The pathogenic potential of the fungus was examined as per Magray et al.^22^ Zoospores were germinated following Tandel et al.^4^. Briefly, 60 numbers of healthy acclimatized fingerlings of *P. hypophthlamus* (22.7 ± 1.8 g) were distributed in six tanks and after 5 days of further acclimatization, skin swabs of 12 fish samples were taken and inoculated onto PDA plates and incubated at 20 ℃ for 48 h. Fish that did not show any fungal growth were used for the pathogenicity study. Freshly grown hyphae with a diameter of about 5 mm were placed on PDA plates and surrounded by sesame seeds as bait. Following 48 h incubation, the seed baits with attached hyphae were taken out and washed twice with PBS on sterile glass slides, and then put in 6-well plates containing APW. Following incubation at 20 C for 18 h, the concentration of zoospores in each well was determined by mixing 100 µL of the APW culture broth with 100 µL of resazurin (HiMedia, India) solution; 10 µL of it was put in a Neubauer haemocytometer and unstained zoospores were counted under a microscope. The viable zoospores (1 × 10^6^ nos. per tank) were added in four inoculation tanks; two more tanks were kept as uninoculated control. Fish were maintained with routine feeding and partial water exchange once a week, and they were observed for two weeks for the development of any illness, skin lesions, or death. Infection patterns and mortalities were recorded in both the groups. The study was conducted in two seasons – winter (January) and summer (May).

### Statistical analysis

A one-way ANOVA with Duncan test was carried out for analysis of in vitro fungal growth at different temperature regimes; two-way ANOVA with Duncan test was performed to examine differences in fungal growth in response to different antifungal compounds at different concentrations and incubation time. All the parameters were compared by Bartlett’s test (*P* < 0.05) and Tukey’s test (*P* < 0.05) and graphs were made using GraphPad Prism 10.2.3 (GraphPad Software, Inc., San Diego).

## Results

### Occurrence of superficial mycosis in freshwater cage culture and fungus isolation

**Fig. 1:**
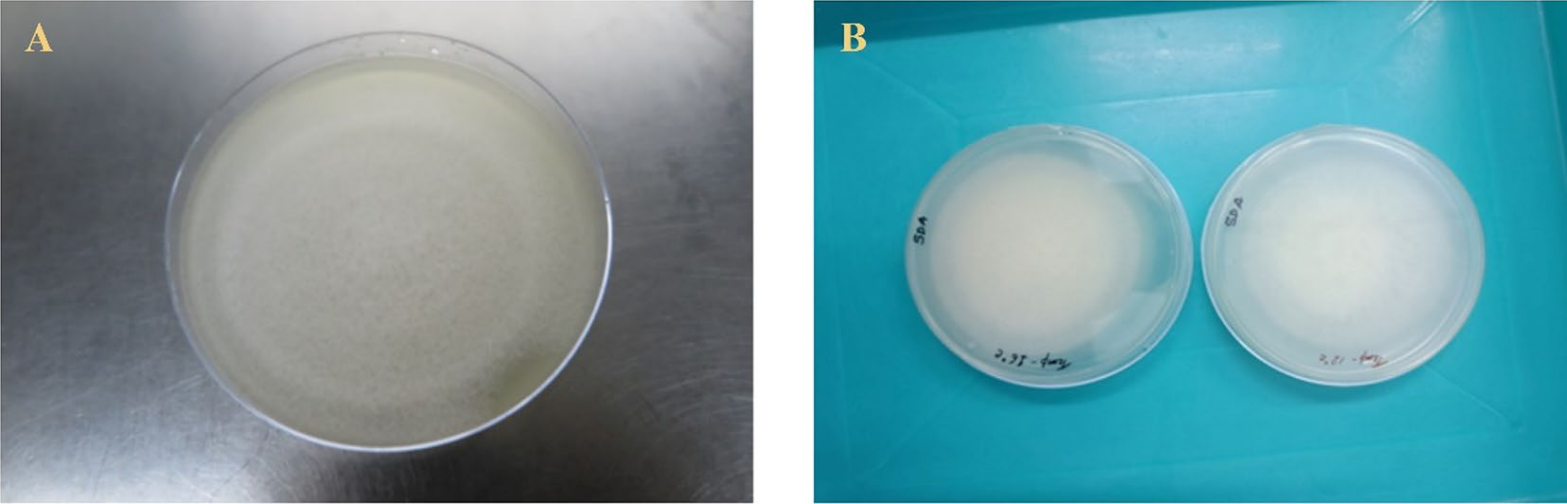
Colony growth characteristics of the isolated fungus on PDA plates. (**A**): growth at 24 h; (**B**): growth at 72–96 h.

A survey in freshwater cage enclosures in Indian reservoirs recorded soft cottonwool-like lesions on fins and occasionally on the tail and body surface of some of the *Pangasianodon hypophthalmus* fingerlings in winter but not in summer months. Upon inoculation of swabs and tissue samples of fins onto different agar media, fungal Growth became visible in 24–36 h, which grew fast to cover the entire plate in 72–96 h (Fig. 1b). Growth was the fastest and most luxuriant in IMA and PYEA, which however, made demarcation of different colonies and thus isolation of fungi in pure culture difficult; the growth was slowest in SDA. PDA gave a moderate rate of growth and was preferred for initial isolation and further studies. Four pure culture fungi isolates were obtained on PDA from 9 diseased fish. On PDA the growing colonies were filamentous, low elevated, and off-white in colour, with hyphae penetrating inside the media and there was presence of a sol-like substance below the colony (Fig. 1).

### Morphological characteristics of the fungus

**Fig. 2:**
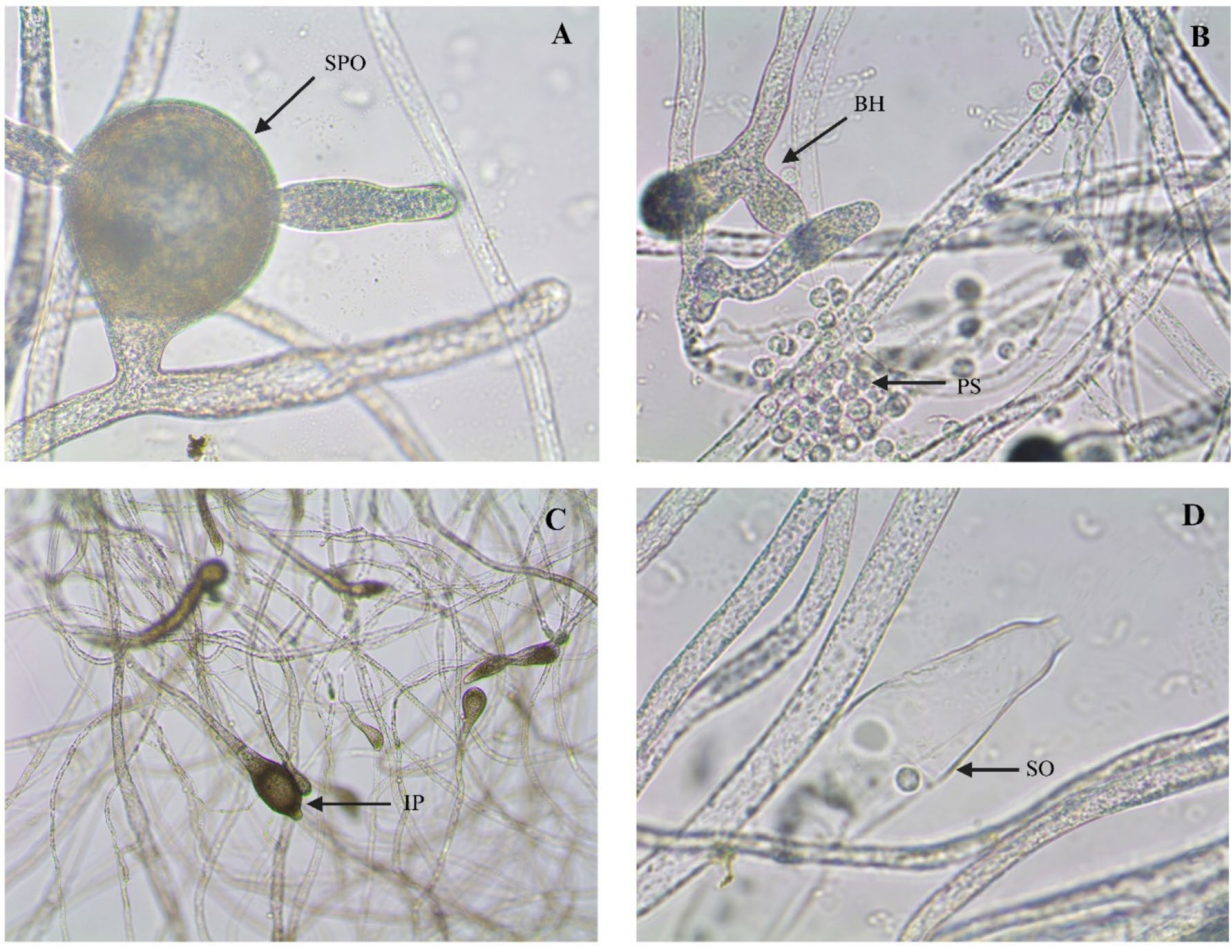
Morphology of *Saprolegnia parasitica* incubated with sesame seed at 20 °C for 18–20 h under a light microscope. (**A**) shows sporangium (SPO); (**B**) shows primary spores (PS) release from the oogonium and branched hyphae (BH) with spores; (**C**) shows immature sporangium intercalary (OI); (**D**) shows elongated hyphae with sub-centric oospores (SO).

**Fig. 3:**
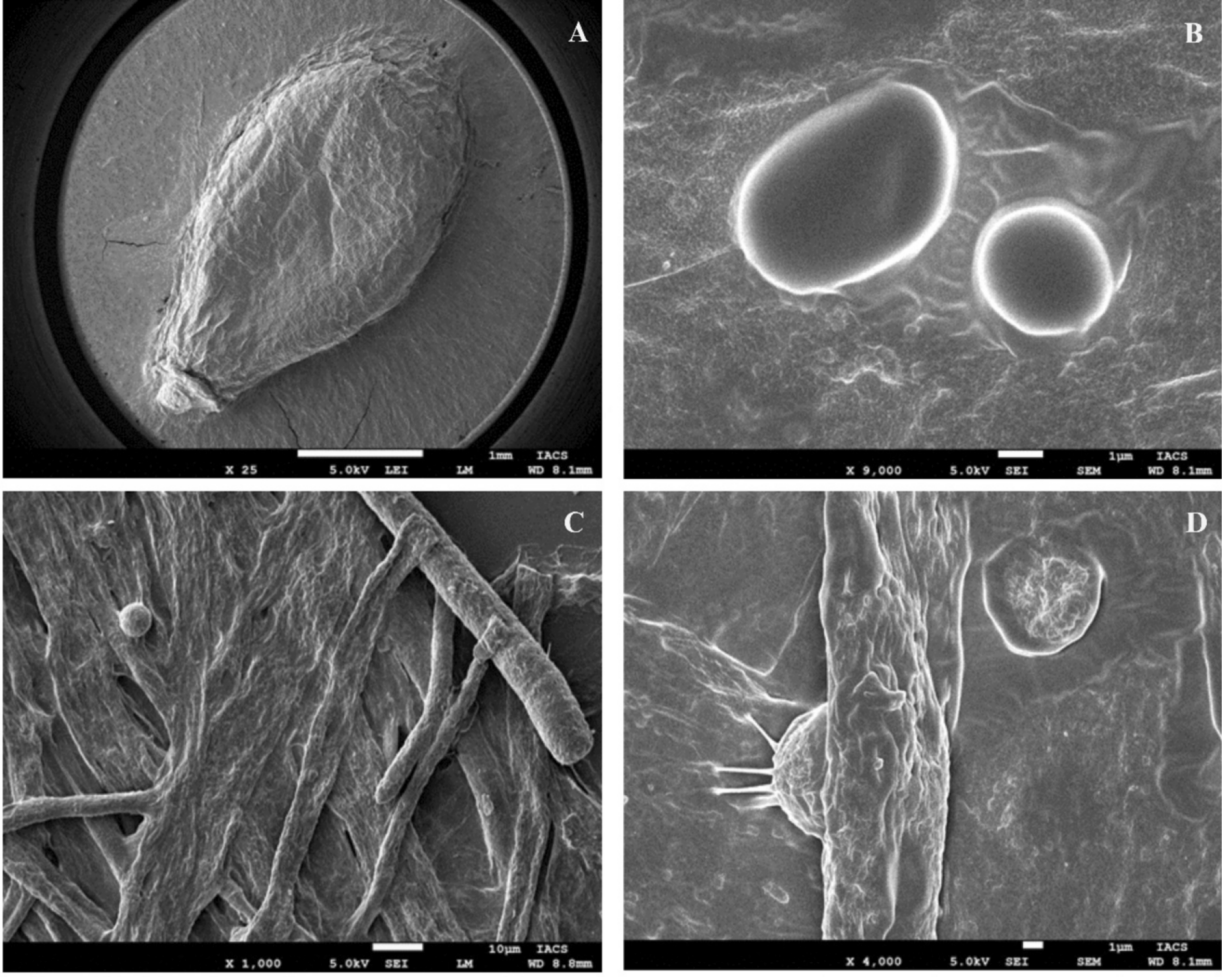
Scanning electron microcopy of *Saprolegnia parasitica* with sessame seed as bait. (**A**): The hyphae of *S. parasiitca* attahced to sesame seed surrounding; (**B**): Primary zooposres; (**C**): Numerous branched hypahe with encysted spores; D: Long hairs on the surface of spores.

Light microscopic examination of the fungal isolates revealed the presence of gemma in abundant numbers, elongated mycelia, non-septate hyphae, zoosporangia, zoospores, and mature zoosporangia containing zoospores (Fig. 2). Sexual characters were not observed in in vitro culture. Based on the morphology the isolates were tentatively identified as *Saprolegnia parasitica*. Electron microscopic observation of the hyphae attached to sesame seed revealed hyphal structures, resting stage of zoospores, cysts with bundles of long hairs with terminal hooks on the cyst surface which is a typical ultrastructure feature of *S. parasitica* (Fig. 3).

### Molecular identification of the fungus

**Fig. 4:**
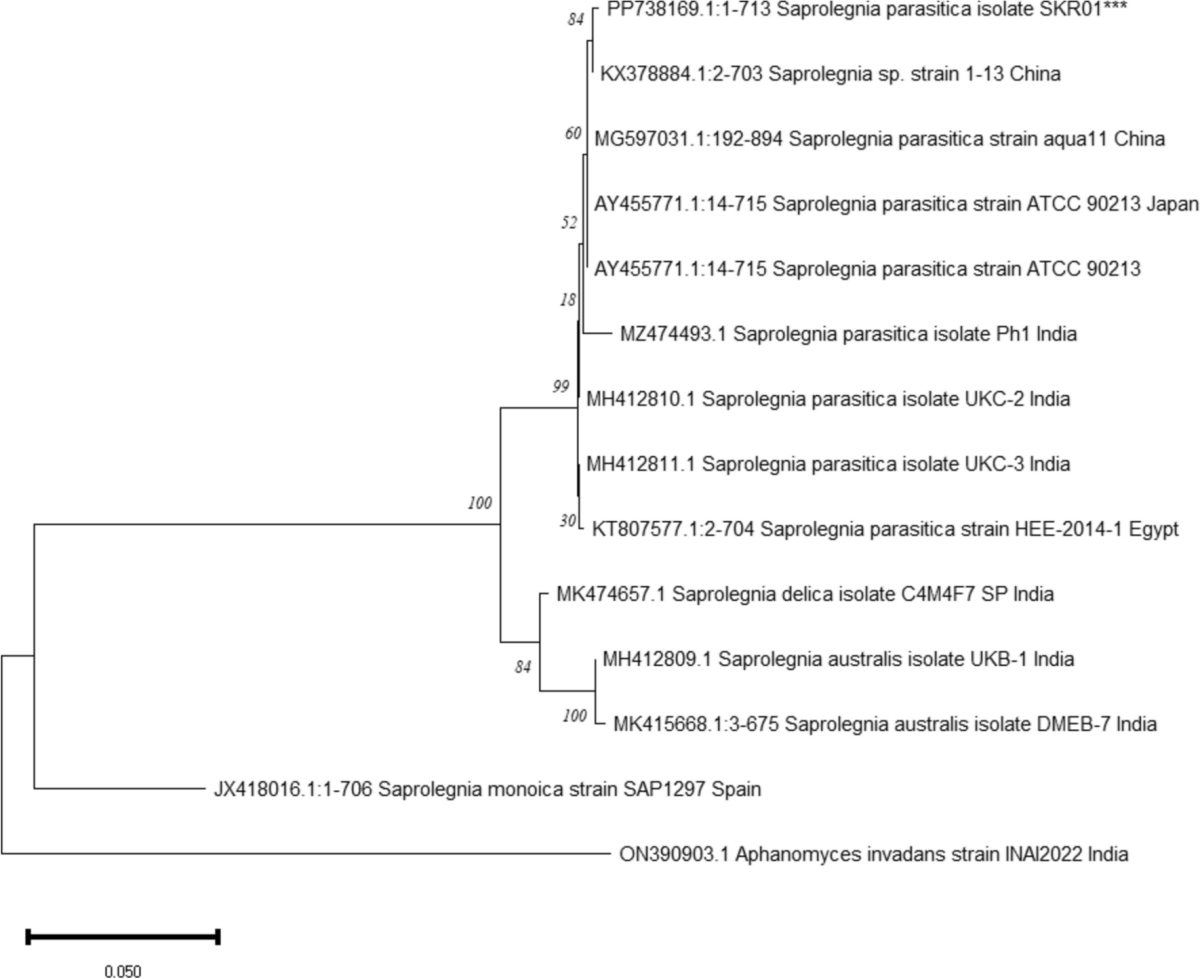
Evolutionary relationship of the present isolate (marked with “***”) with other *Saprolegnia* species and other *S. parasitica* strains. The phylogenetic tree was constructed following maxiumum likelihood method using MEGA 11.0 and boot strapped 1000 times to assess reliability of the data. Numbers next to the branches indicate bootstrap values and scale bar represents evolutionary distance.

PCR amplification of the internal transcribed spacer (ITS) region generated 750 bp long amplicons. Sequencing of the amplicons of different isolates yielded identical nucleotide sequences, which showed more than 99% similarity with other published *S. parasitica* sequences in NCBI BLASTn search. A representative sequence has been submitted to the NCBI GenBank with Accession No. PP738169.1 which formed a clade with other *S. parasitica* sequences in the phylogenetic tree (Fig. 4).

#### Optimum temperature requirements for growth of *S. parasitica*

**Fig. 5:**
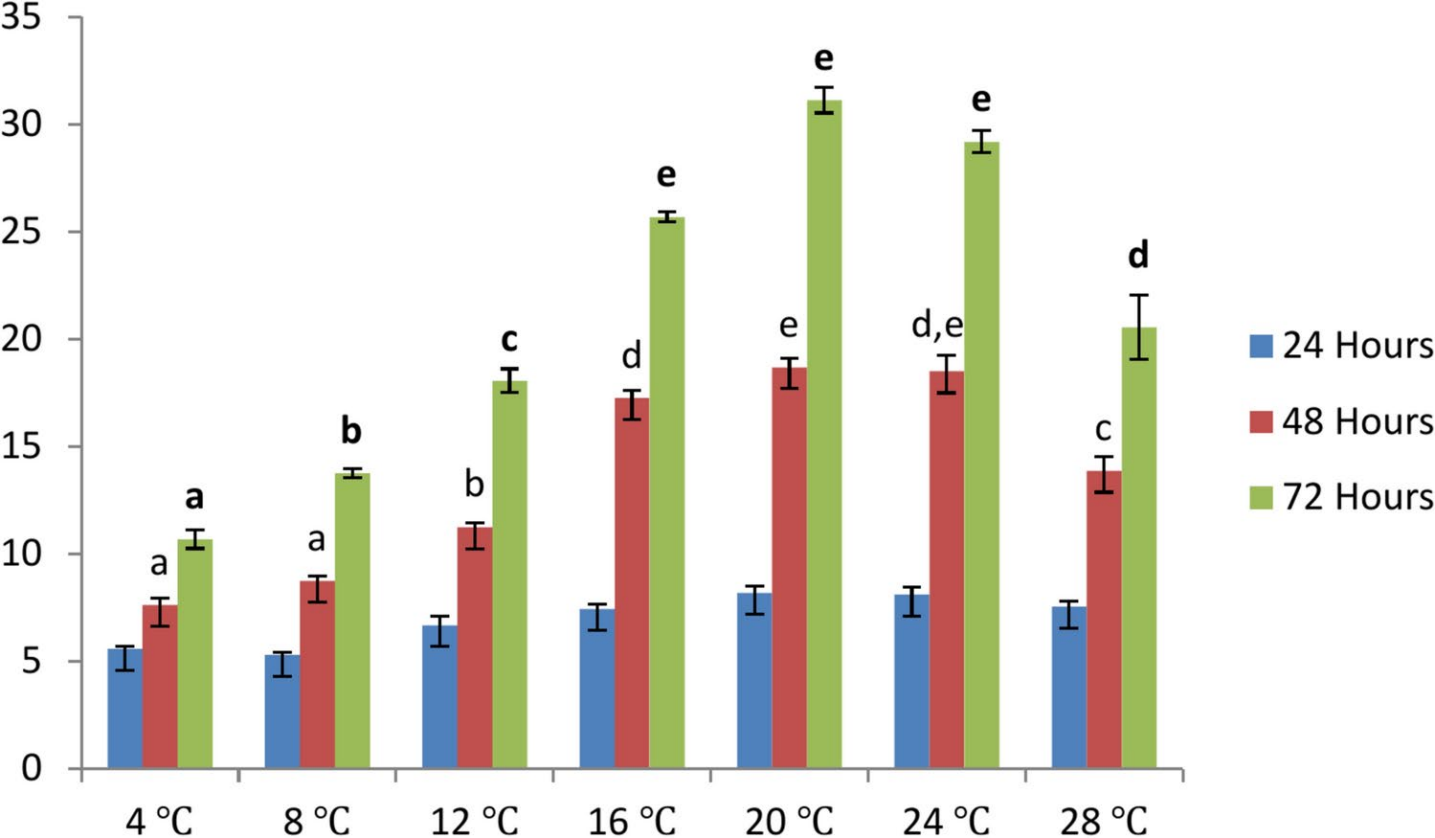
Growth of *S. parasitica* at different temperatures. Fungal growth at 48 and 72 h incubation were compared for statistical significant difference at 5% level. Different non-bold letter superscripts indicate significant difference at 48 h growth; different bold letter superscripts indicate significant difference at 72 h growth.

A study of the growth of the isolates at different temperatures, from 4 to 28 °C, showed a fast increase in colony size with incubation time, with maximum growth attaining at 20 ℃. There was no hyphal growth at 4 °C, poor growth at 8 °C, and an increase in growth till 20 ℃, followed by a decline at higher temperatures (Fig. 5).

### Experimental pathogenicity of *S. parasitica* in *P. hypophthlamus*

**Fig. 6:**
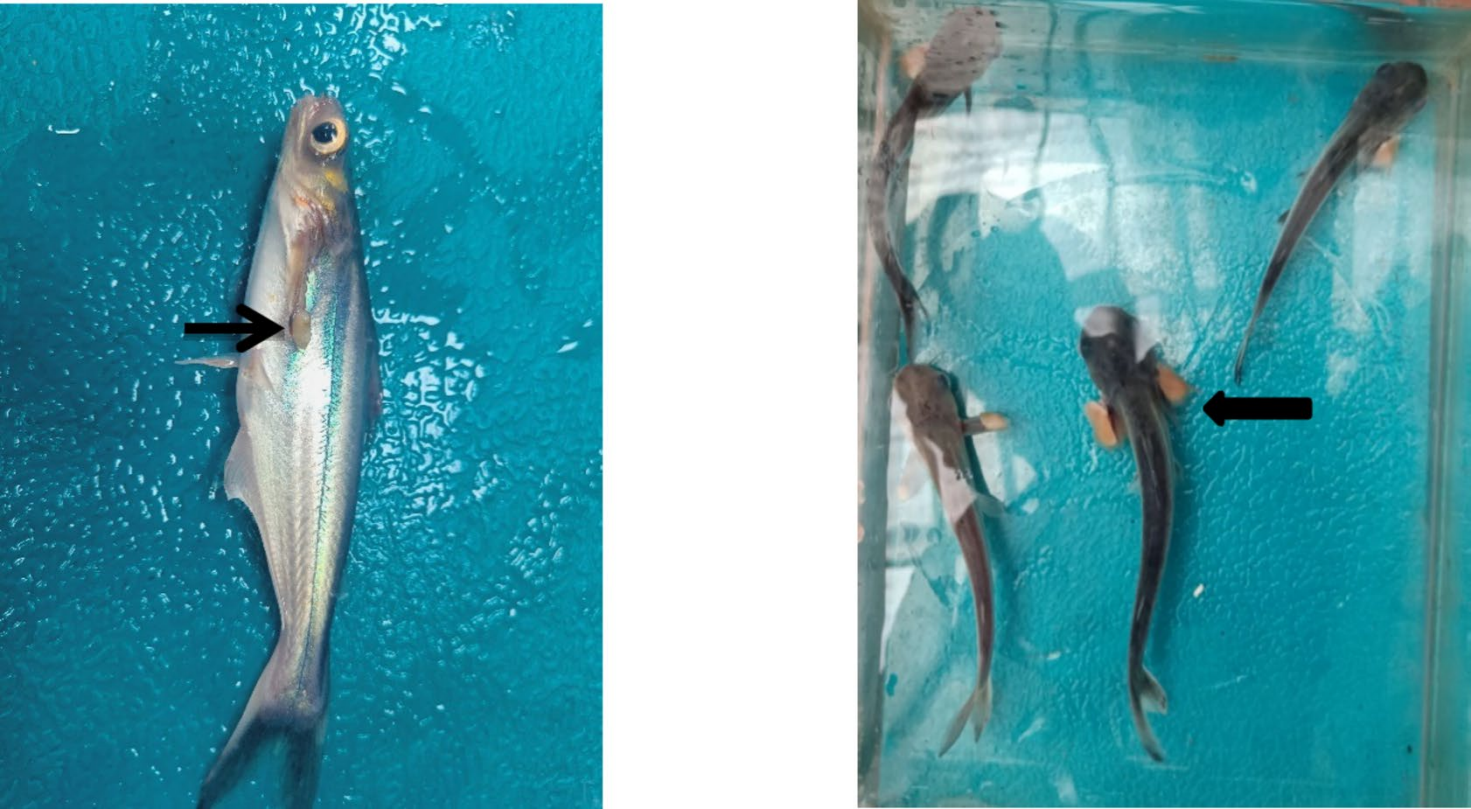
The zoospores of *Saprolegnia parasitica* induced disease following experimental infection. The lesion is most visible on pectoral fins (arrow).

Bioassay using fungal zoospores as an indefinite immersion challenge in Pangasius in January (water temperature 14 ± 1.5 ℃) resulted in the development of fungal growths on fins and tail after 4–5 days of spore addition and 70–80% mortality in experimental tanks while no fish in un-inoculated tanks developed such lesion. However, the study repeated in May (water temperature 31 ± 1.8 ℃) failed to induce clinical lesions or mortality in any of the challenged fish (Fig. 6).

#### MIC of antifungal compounds against *S. parasitica* spores

**Fig. 7:**
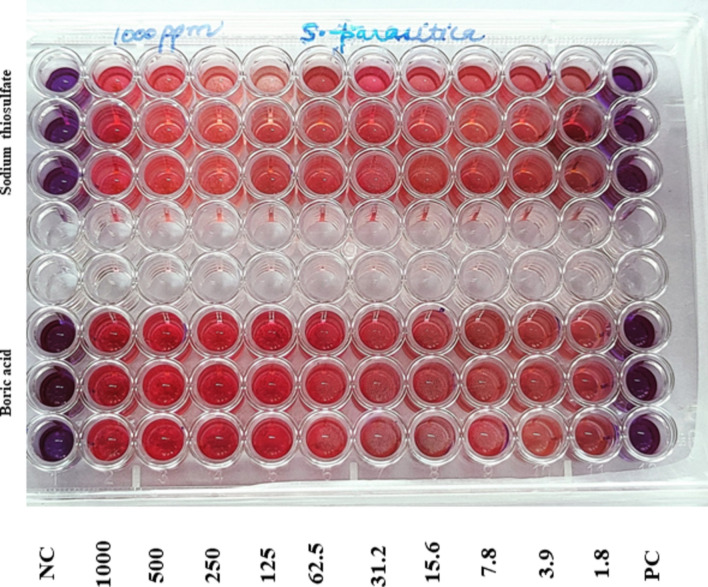
Microtitre plate showing growth inhibition of fungal spores by graded concentrations of boric acid (upper 3 rows) and sodium thiosulfate (bottom 3 rows). The lowest concentrations of a test compound giving no colour change of resazurin were taken as its minimum inhibitory concentration (MIC). NC: Negative control; PC: Positive control (malachite green).

A study on the susceptibility of the fungal spores to selected chemicals and pharmacologically active substances revealed malachite green as the most effective chemical against the fungus with MIC of 2 mgL^−1^. Clotrimazole also inhibited the spore growth at 2 mgL^−1^. In contrast, fluconazole, potassium permanganate, and sodium thiosulfate did not show any inhibitory activity even at 250 mgL^−1^ (Fig. 7). There was no significant (*P* < *0.05*) growth difference, as compared to control when different concentrations of boric acid were tested. However, copper sulfate prevented growth of the fungus at 62.5 mgL^-1^ and higher concentrations.

#### MIC of different compounds against *S. parasitica* hyphae

**Fig. 8:**
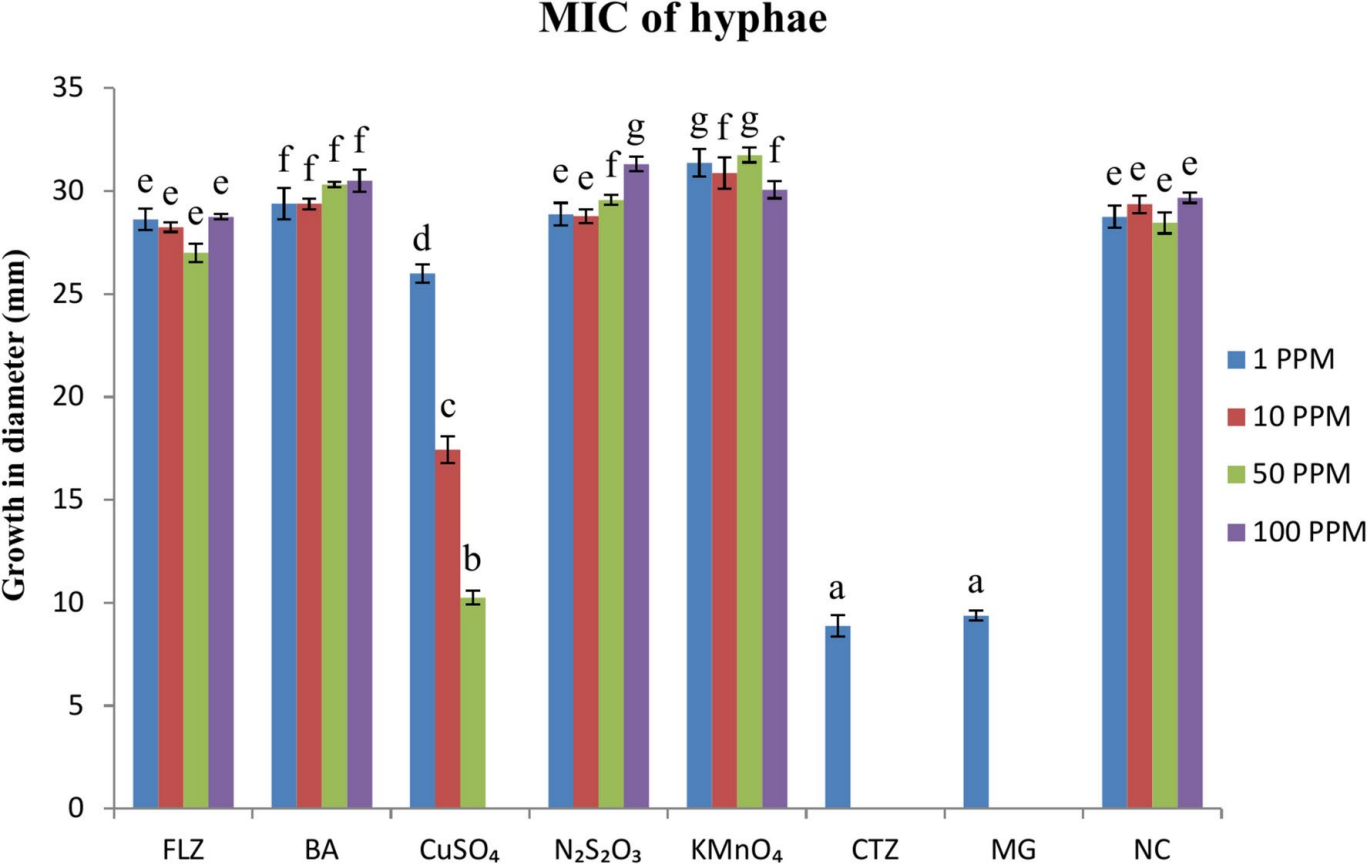
MIC of different antifungal chemicals and drugs against *S. parasitica* hyphae incubation periods for 72 h at 20 °C. Different superscripts indicate significant difference at a 5% level.

Like in case of spores, clotrimazole and malachite green caused partial inhibition of hyphal growth at 1 mgL^−1^ and complete inhibition at 2 mgL^−1^ concentrations. Copper sulfate caused partial growth inhibition at 50 mgL^−1^, and complete growth inhibition at 100 mgL^−1^ (Fig. 8). Fluconazole, sodium thiosulfate, boric acid and potassium permanganate did not prevent growth of the fungal hyphae even at 100 mgL^−1^.

## Discussion

Saprolegniasis is one of the most common diseases infecting several freshwater fish species, and fish hatcheries^23^. Although *P. hypophthalmus* is considered a hardy fish, the present study recorded 11.7% of catfish mortality in freshwater cage culture systems in winter months. The disease prevalence, severity of infection and mortality increased with the progression of winter, threatening the survival of standing crop in cages in winter months. Kumar et al.^25^ reported a wide range of catfish mortality ranging from less than 5% to 100% in the winter season in different ponds in Uttar Pradesh, India. In laboratory bioassay, the fungus induced a high level of mortality in winter but failed to induce any disease in summer, establishing high susceptibility of the catfish to the fungus in colder months, likely due to low temperature-induced immunosuppression. Besides influencing the host’s susceptibility, the present study showed the strong influence of ambient temperature on the fungal growth – luxuriant growth of the fungus was observed at 20 °C, which might easily disrupt the delicate host–pathogen homeostasis in favour of the pathogen, especially when the fish is already immunocompromised. There was no hyphae growth at 4 °C, which corroborated with the lower temperature limitation of the fungus described by Tandel et al.^4^ and Magray et al.^22^ but contrasts with the finding of Liu et al.^24^ from China. This difference might originate from the temperature regime of the geographical area under study, nutrient load in the ecosystem, and water quality, which might significantly influence the biology of aquatic microorganisms. Further, the fungus growth decreases above 20 ℃, explaining little occurrence of saprolegniasis in summer months. *S. parasitica* is considered eurythermous, however, several researchers have recorded the infection at lower water temperatures. It remains to be seen whether temperature also influences expressions of virulence factors such as lectins, peptides, haemolysin, toxins, protease, etc. Kitancharoen et al.^25^ reported good growth of *S. parasitica* from visceral mycosis at 30 ℃ and the production of more motile zoospores than the isolates from superficial lesions.

Based on morphology and molecular evidence, the causative agent of superficial mycosis was identified as *S. parasitica,* the most prevalent and pathogenic oomycete pathogen in India. In vitro, the fungus reproduced asexually, and sexual form was not observed. Morphological features such as gemmae, elongated mycelia, zoosporangia, zoospores, and branched hyphae with immature sporangia and cysts observed here have also been reported earlier for *S. parasitica* from Pangasius from Vietnam and India^3,26^, snow trout from India (Tandel et al.^19^, common carp from Iraq^27^, and brown trout from United Kingdom^16^. The fungus follows asexual reproduction to parasitize the host surface through secondary cysts. Bundles of long hooked hairs on the cyst surface, observed at higher magnifications, might facilitate adhesion of the cyst on fish skin and thus enhance the pathogenic potential of *S. parasitica* over other *Saprolegnia* spp.^15,20^.

To date, there is no specific and compelling preventive or therapeutic intervention to combat fungal infections in fish. There have been meagre efforts to develop cost-effective control measures for fungal diseases of fish^19^. Traditionally, formaldehyde and malachite green have been the disinfectants of choice for treating oomycete infections in fish; however, they are either banned or are recommended for use with utmost caution owing to their carcinogenic and toxicological properties^28,29^. Recently, the potential of combined dietary incorporation of β-1,3 glucan and fructooligosaccharides in immunomodulation has been demonstrated^30^. However, using such functional molecules in fungal disease control has yet to reach field-level application. Imidazole and triazole groups of drugs are the mainstay of treatment of fungal infections in humans and animals but are meagrely studied against fish fungi. Fluconazole, sodium thiosulfate, and boric acid have been studied for their antifungal activity in laboratory conditions^2,18,31,32^ with mixed success. The present study also observed little antifungal activity of potassium permanganate, sodium thiosulfate, and boric acid against *S. parasitica.*

In comparison, malachite green, a banned chemical for aquaculture use, completely inhibited zoospore production at concentrations as low as 2 mgL^−1^. Copper sulfate, commonly used to treat fungal diseases in field conditions, inhibited the growth of zoospores and hyphae at 62 and 100 mgL^-1^, respectively, indicating its low efficacy against the fungus; further, it has substantial toxicity in fish as well as in phytoplankton limiting its field use. In Indian aquaculture, the use of fluconazole has been recorded, especially in Pangasius culture tanks^33^, despite the lack of any study on the efficacy of the drug against fungal diseases in catfish. In-feed administration of fluconazole at a dose rate of 10–30 mgkg^-1^ for 45 days offered 81.8–90% survival of L*. rohita* against experimental infection of *S. parasitica*^34^. In contrast, the present study found little activity of fluconazole against *S. parasitica *in vitro*.* Similar low activities of epoxiconazole, fluconazole, itraconazole, and posaconazole against *Saprolegnia* have been reported^7^, suggesting low activity of triazole compounds against the fish fungus. Hopefully, the present study detected clotrimazole as a potent compound in inhibiting the germination of zoospores and growth of *S. parasitica* hyphae at a very minimum concentration. Considering the recent growth trajectory of Pangasius production and vulnerability of the fish species to saprolegniasis clotrimazole might be a saviour for catfish farmers. However, its in vivo efficacy, safety, and pharmacokinetic aspects need to be studied before recommendation for any larger trial.

## Conclusion

The present study identifies *Saprolegnia parasitica* as a pathogen of aquaculturally important catfish *P. hypophthalmus*. Low ambient temperature favoured the growth of the fungus, enabling it to precipitate winter outbreaks. Among the well-known antifungal compounds, only clotrimazole had shown significant inhibitory activity against the fungus, making it a potential VMP for controlling superficial mycosis in the catfish. This information serves as a baseline for developing preventive or therapeutic interventions to control saprolegniasis and reduce biological loss.

### Ethical statements

The experimental work using *Pangasianodon hypophthalmus* was approved by Institutional Ethics Committee of ICAR-Central Inland Fisheries Research Institute, Kolkata, India (approval no 2167/GO/RBi/S/22/CPCSEA/03/2022–23) and were conducted following guidelines and regulations of Institutional Biosafety Committee (IBSC), Department of Biotechnology (DBT) and Ministry of Science and Technology, Government of India Rules and ARRIVE.

## Supplementary Information

Supplementary Information.

## Data Availability

The authors confirm that the data supporting the findings of the study are available within the article.

## Abbreviations

PDA: Potato dextrose agar
VMP: Veterinary medicine products
CTZ: Clotrimazole
FLZ: Fluconazole
MIC: Minimum inhibitory concentration
PCR: Polymerase chain reaction
APW: Autoclaved pond water
PBS: Physiological buffer saline
