# Supplementary material for: Isolation and characterization of Saprolegnia parasitica from cage-reared Pangasianodon hypophthalmus and its sensitivity to different antifungal compounds
Source: Sci Rep. 2024 Dec 28;14:30720. doi: 10.1038/s41598-024-80075-0 (PMC11681207; doi:10.1038/s41598-024-80075-0)
Supplement: Supplementary file 1 — Supplementary Information. [file 41598_2024_80075_MOESM1_ESM.docx]

| Supplementary file 1: The cumulative mortality pattern following experimental infection of Pangasius |
| --- |
